# Sodium channel point mutations associated with pyrethroid resistance in Chinese strains of *Culex pipiens quinquefasciatus* (Diptera: Culicidae)

**DOI:** 10.1186/1756-3305-7-369

**Published:** 2014-08-15

**Authors:** Minghui Zhao, Yande Dong, Xin Ran, Xiaoxia Guo, Dan Xing, Yingmei Zhang, Ting Yan, Xiaojuan Zhu, Jianxin Su, Hengduan Zhang, Gang Wang, Wenjun Hou, Zhiming Wu, Chunxiao Li, Tongyan Zhao

**Affiliations:** Beijing Institute of Microbiology and Epidemiology, State Key Laboratory of Pathogen and Biosecurity, Beijing, China; Anhui Medical University, Hefei, China

## Abstract

**Background:**

Pesticide resistance due to sodium channel point mutations has been well documented in many mosquito species.

**Methods:**

We tested the resistance of six, wild, Chinese populations of the mosquito *Culex pipiens quinquefasciatus* to deltamethrin and cyhalothrin. The full length of the sodium channel gene was cloned and sequenced from a pooled sample of mosquitoes from each population.

**Results:**

Seven amino acid alterations were found (V250M, R436K, M943V, I973T, L1035F, L1035S and E1901D). Correlation between the frequencies of these mutations and the level of pesticide resistance (LC_50_) associated with them indicates that those at position L1035 (corresponding to position L1014F in the house fly, *Musca domestica*; GenBank Accession No.: X96668) are associated with resistance to deltamethrin and cyhalothrin. The frequency of the L1035F mutation was significantly correlated with resistance to deltamethrin (R^2^ = 0.536, P = 0.049) and cyhalothrin (R^2^ = 0.626, P = 0.030), and the combined frequency of the L1035F and L1035S mutations was significantly correlated with resistance to both deltamethrin (R^2^ = 0.661, *P* = 0.025), and cyhalothrin (R^2^ = 0.803, *P* = 0.008). None of the other mutations were correlated with either deltamethrin or cyhalothrin resistance. Interestingly, an HWE test indicated significant linkage between the M943V and I973T mutations (*P* < 0.01), but further research is required to determine the biological significance of this linkage.

**Conclusions:**

Identifying these mutations may be of practical benefit to the development of pesticide resistance management programs.

## Background

*Cx. pipiens quinquefasciatus* is the predominant mosquito species in urban environments in southern China. This species is a major biting nuisance, particularly in urban areas where it thrives in wet pit-latrines, polluted puddles, gutters, and blocked open drains. It is also a major vector of filariasis [1], West Nile virus (WNV) [2], St. Louis encephalitis virus (SLEV) [3], and Rift Valley Fever virus (RVFV) [4, 5]. *Cx. p. quinquefasciatus* is one of the most studied mosquito species with respect to insecticide resistance.

“Pyrethroid” is the general term for a group of synthetic chemicals based on the structure of natural pyrethrins derived from Chrysanthemum flowers [6]. Pyrethroids are now becoming the most commonly used insecticides for mosquito control [7]. Compared to other insecticides, most pyrethroids are nontoxic to mammals but have a high knockdown effect on insects. Unfortunately, the extensive use of pyrethroids has led to the widespread development of resistance to this group of pesticides in mosquito populations [8–10]. The increase in resistance can be both large and rapid; one study reported an approximately 298-fold increase in resistance in *Cx. p. quinquefasciatus* after 10 generations of exposure to deltamethrin [11].

Modification of the active site of the sodium channel is an important mechanism involved in pyrethroid resistance [12, 13]. Voltage-gated sodium channels are integral transmembrane proteins responsible for the rapidly rising phase of action potentials that is critical for electrical signaling in most excitable cells. Because of their crucial role in membrane excitability, sodium channels are the target of a great variety of neurotoxins, including insecticidal pyrethrins [14]. Pyrethroid insecticides bind to insect para-sodium channels locking them in an open state thereby disrupting neuronal signaling resulting in paralysis and death.

Point mutations in the para-sodium channel gene that make sodium channel insensitive to pyrethrins are an important mechanism of resistance to pyrethroid insecticides in various insect species [15–22]. The most common mutation is the substitution of leucine by phenylalanine at residue L1014, commonly referred to as the kdr (knockdown resistance) mutation. Resistance conferred by the L1014F mutation was first described in the Italian housefly with respect to DDT [23] and subsequently named knockdown resistance [24]. Variants of this mutation such as L1014S, L1014H, L1014W and L1014C have also been reported [25–28]. Another mutation, M918T, has also been found in many insects [29–31]. This mutation and the L1014F mutation often occur together in which case they confer what has been called “super-kdr resistance”.

One approach to investigating pesticide resistance in mosquitoes is to first identify mutations that could potentially confer resistance and then attempt to correlate the frequency of these with actual pesticide resistance. Through a combination of insecticide bioassays and molecular techniques, we identified mutations associated with resistance to deltamethrin and cyhalothrin in six wild Chinese populations of *Cx. p. quinquefasciatus* and assessed the similarity between these and previously documented mutations in the sodium channel gene. Knowledge of these mutations may have practical benefits for further research on pesticide resistance in mosquitoes and for designing resistance management programs.

## Methods

### Mosquito strains

**Figure 1:**
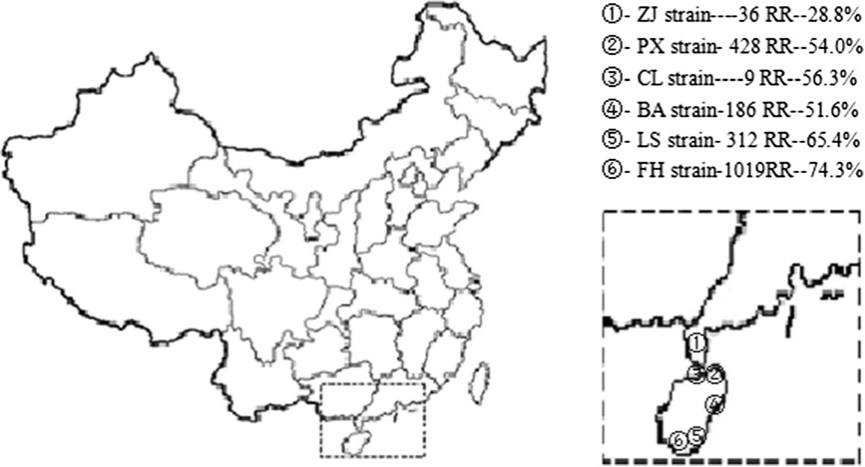
**The distribution of collecting sites of** ***Cx. p. quinquefasciat*** **us.** ①-⑥ indicated the six field strains of *Cx. p. quinquefasciat*us in China. RR was resistance ratio to deltamethrin, and the percentages were mutation frequencies of L1035F.

*Cx. p. quinquefasciatus* larvae and some female adults were collected from six different field locations: Haikou Poxiang (E110°19′33.79″, N19°59′55.07″), Haikou Changliu (E110°11′50.36″, N20°0′50.25″), Qionghai Boao (E110°34′57.13″, N19°09′42.07″) and Sanya Fenghuang (E109°26′54.38″, N18°18′2.91″) in 2012; Guangdong Zhanjiang (E110°23′53.05″, N21°11′30.19″) and Hainan Lingshui (E110°2′15.01″, N18°30′21.77″) in 2013 (Figure 1). Larvae were reared to adulthood in captivity but the wild caught female adults were frozen in liquid nitrogen for subsequent genetic testing. The control population for insecticide bioassays was a laboratory strain that had not been exposed to insecticides for more than 10 years.

### Bioassay

Bioassays were conducted by putting thirty late 3rd or early 4th instar larvae into pans containing 199 ml water and 1 ml of either deltamethrin or cyhalothrin. The insecticides were added to each pan using an automatic pipette according to the methods specified by the WHO [32]. Larval mortality was recorded 24 h after each treatment. No food was offered to the larvae during bioassays. Larvae were kept in a laboratory under the following conditions: 14 L:10D photoperiod, 75% relative humidity and a temperature of 26 ± 1°C during bioassays. Bioassays of each insecticide were repeated three times. Statistical analyses were performed using SPSS software version 13.

### Extraction of RNA, cDNA synthesis and PCR amplification

**Figure 2:**
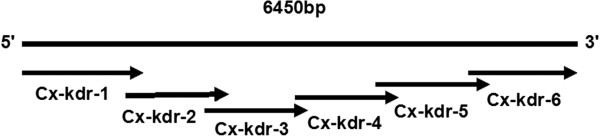
**Schematic diagram of amplification of the sodium channel gene of** ***Cx. p. quinquefasciatus*** **.** The complete gene sequence was 6450 bp, and the gene’s six sections are indicated by arrows.

Total RNA was extracted from mosquitoes from each population with Trizol reagent (GBT) following the manufacturer’s protocol and cDNA was synthesized from the extracted total RNA with a cDNA synthesis kit (Transgen Biotech). The synthesized cDNA was stored at -20°C. Gene-specific primers from Zhao et al. (2014) used to amplify the sodium channel gene of specimens from each population [33]. The sodium channel gene is 6450 bp and is divided into six sections (Figure 2).

### Cloning and sequencing of PCR products

To identify mutations in the sodium channel gene, the cDNA of a pooled sample of *Cx. p. quinquefasciatus* comprised of specimens from each of the six populations was cloned and sequenced. PCR products were purified using a universal DNA purification kit (TIANGEN) and the purified products were ligated into the pEASY-T1 vector (TRANSGEN). The recombinant plasmids were then cloned into Trans1-T1 competent cells (TRANSGEN). The microbials were spread on LB solid medium (including ampicillin, X-gal, IPTG) and cultured overnight. White clones were selected, placed in LB liquid medium and cultured to turbidity. Positive clones were identified by PCR using M13 forward and reverse primers and sequenced by Tianyi Biotech [34]. Based on the discovery of clones, the allele frequency of each mutation was determined by specific PCR amplification and sequencing of the mutations found in each population. In this procedure, a single mosquito’s RNA was extracted and reverse transcribed to cDNA, then amplified by specific PCR before being sequenced. The gene-specific primers used to amplify the mutations in the *Cx. p. quinquefasciatus* para-sodium channel gene were designed in NCBI Primer BLAST (Table 1).

**Table 1:** **The four pairs of specific primers used to amplify sodium channel gene mutations detected in** ***Cx. p. quinquefasciatus***

Primers	5′ → 3′Sequence	length (bp)
Cx-M1	GATGATCATGCCATCCACGC	548
	CTTCCTCACATTGGCCAGCA	
Cx-M2	CGACAGTCGAATCTACCGAGGTG	995
	CTTCTCCTTGTTGTTGTCGTCG	
Cx-M3	GAGCTGTTCATCACGCTCTG	689
	GCCGACAAACTCGAGGAACC	
Cx-M4	GCCGGATAACGACAAGGGTT	661
	GGGCTCGTTATCACAGGCAT	

### Analysis of genetic linkage between mutations

We use the GENEPOP software package to estimate conformity to the HWE and genetic linkage between mutations.

### Correlation of pesticide resistance with the frequencies of different mutations

The resistance (LC_50_) of the six populations to deltamethrin and cyhalothrin was determined by bioassay and the allele frequencies of the various mutations determined by gene-specific amplification and sequencing as described above. The LC_50_ of a laboratory strain that had not been exposed to either pesticide was also determined to serve as a control. Correlations between resistance and mutation frequency were analyzed using Graphpad Prism 5.

## Results

### Resistance to deltamethrin and cyhalothrin

LC_50_ values of the seven different populations ranged from 0.009 to 1.019 ppm for deltamethrin and from 0.014 to 1.291 ppm for cyhalothrin (Table 2). The CL strain was the most susceptible to both insecticides. The FH strain was the most resistant; 1019 times more resistant to deltamethrin and 1291 times more resistant to cyhalothrin than the laboratory strain (LC_50_ 0.001 ppm).

**Table 2:** **Levels of deltamethrin and cyhalothrin resistance measured in** ***Cx. p. quinquefasciatus***

Population	Insecticide	LC _50_ (ppm) (95% CL) ^1^	Regression Equation	χ2	***P-*** value	RR ^2^
LA	deltamethrin	0.001 (0.001, 0.001)	Y = 7.412 + 2.371x	29.78	0.019	1
	cyhalothrin	0.001 (0.001, 0.001)	Y = 6.819 + 2.356x	16.76	0.606	1
CL	deltamethrin	0.009 (0.006, 0.011)	Y = 3.028 + 1.475x	13.34	0.648	9
	cyhalothrin	0.014 (0.010, 0.017)	Y = 2.898 + 1.551x	10.63	0.832	14
PX	deltamethrin	0.428 (0.385, 0.476)	Y = 0.842 + 2.284x	31.44	0.175	428
	cyhalothrin	0.396 (0.332, 0.468)	Y = 0.610 + 1.517x	24.84	0.305	396
BA	deltamethrin	0.186 (0.151, 0.231)	Y = 1.314 + 1.799x	23.34	0.105	186
	cyhalothrin	0.215 (0.172, 0.264)	Y = 0.761 + 1.139x	23.28	0.386	215
FH	deltamethrin	1.019 (0.811, 1.268)	Y = -0.012 + 1.439x	32.20	0.075	1019
	cyhalothrin	1.291 (1.051, 1.560)	Y = -0.165 + 1.484x	16.83	0.396	1291
ZJ	deltamethrin	0.036 (0.031, 0.041)	Y = 3.073 + 2.127x	18.05	0.703	36
	cyhalothrin	0.042 (0.037, 0.048)	Y = 3.048 + 2.215x	26.82	0.218	42
LS	deltamethrin	0.312 (0.234, 0.413)	Y = 0.630 + 0.541x	40.04	0.011	312
	cyhalothrin	0.641 (0.520, 0.800)	Y = 0.221 + 0.497x	25.05	0.295	641

(LA = Lab strain; CL = Haikou Changliu; PX = Haikou Poxiang; BA = Qionghai Boao; FH = Sanya Fenghuang; ZJ = Guangdong Zhanjiang; LS = Hainan Lingshui.

^1^CL = confidence limits, ^2^RR = Resistance Ratio).

### Sodium channel gene mutations

**Figure 3:**
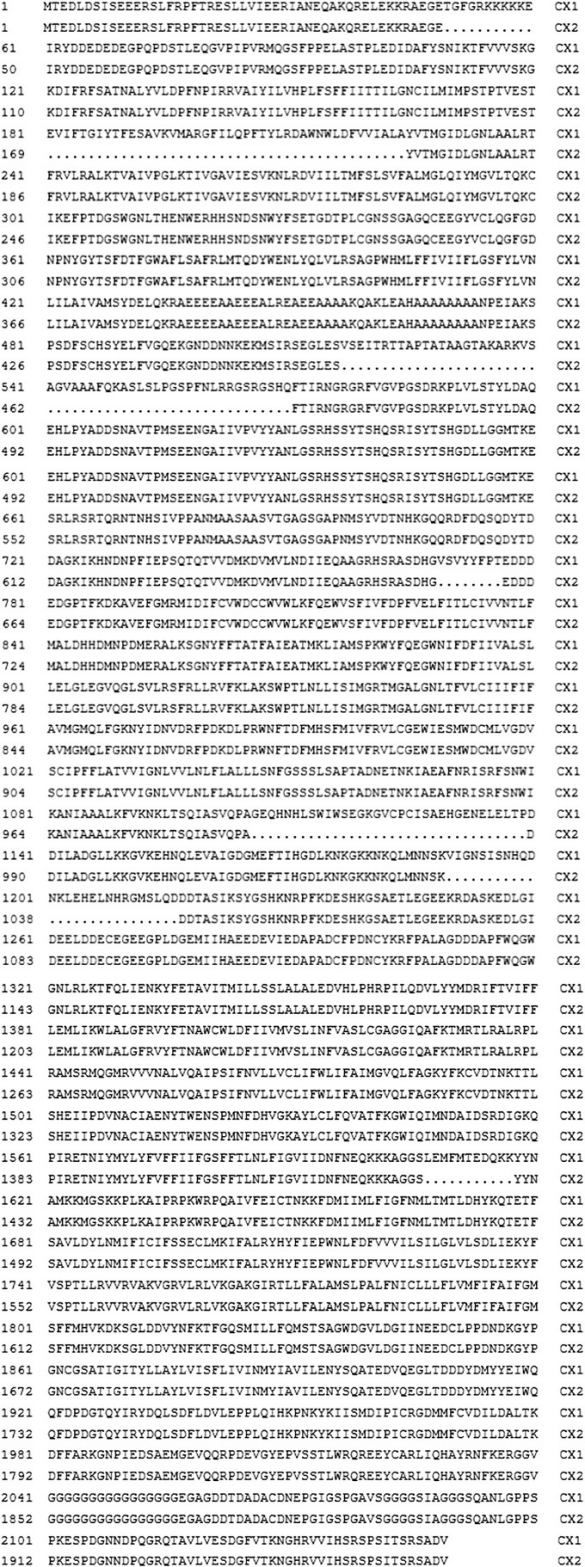
**Alternative splicing in the sodium channel gene of** ***Cx. p. quinquefasciatus.*** CX1 was the sequence of *Cx. p. quinquefasciatus* (GenBank Accession No.: AB453977.1). CX2 was the sequence of *Cx. p. quinquefasciatus* in China. The dots indicated the alternative splicings. In the 517–570, there were three kinds of alternative splicings (517–550, 542–550, and 542–570); In the 1106–1139, there were also three kinds of alternative splicings (1106–1118, 1106–1128, and 1106–1139).

Seven mutations (V250M, R436K, M943V, I973T, L1035F, L1035S and E1901D) were identified. Because these seven mutations were identified in a pooled sample they did not necessarily occur in the same individuals. The seven mutations were V250M: GTG → ATG, R436K: AGG → AAG, M943V: ATG → GTG, I973T: ATC → ACA, L1035F: TTA → TTT, L1035S: TTA → TCA, and E1901D: GAG → GAT. In addition, eleven alternative splices in the sodium channel gene were detected (Figure 3).

### Genetic and genotypic frequencies

Genetic and genotypic frequencies were showed in Table 3.

**Table 3:** **Gene and genotype frequencies of seven sodium channel gene mutations calculated in six populations of** ***Cx. p. quinquefasciatus***

Mutation	Population	n	Genotype frequency and gene frequency (%)			
			SS ^1^	RS ^2^	RR ^3^	R ^4^
V250M	FH	49	61.2	34.7	4.10	21.4
	PX	31	41.9	48.4	9.70	33.9
	BA	29	55.2	37.9	6.90	25.9
	ZJ	31	48.4	51.6	0.00	25.8
	CL	32	65.6	34.4	0.00	17.2
	LS	36	61.1	27.8	11.1	25.0
R436K	FH	27	7.40	92.6	0.00	46.3
	PX	12	0.00	100	0.00	50.0
	BA	10	0.00	100	0.00	50.0
	ZJ	20	0.00	100	0.00	50.0
	CL	13	0.00	100	0.00	50.0
	LS	15	6.70	93.3	0.00	46.7
M943V	FH	32	34.4	65.6	0.00	32.8
	PX	38	44.7	55.3	0.00	27.6
	BA	31	32.3	67.7	0.00	33.9
	ZJ	33	48.5	51.5	0.00	25.8
	CL	32	28.1	71.9	0.00	35.9
	LS	31	16.1	83.9	0.00	41.9
I973T	FH	32	46.9	53.1	0.00	26.7
	PX	38	39.5	60.5	0.00	30.3
	BA	31	29.0	71.0	0.00	35.5
	ZJ	33	51.5	48.5	0.00	24.2
	CL	32	37.5	62.5	0.00	31.3
	LS	31	32.3	67.7	0.00	33.9
L1035F	FH	35	0.00	11.4	68.6	74.3
	PX	38	5.26	18.4	44.7	54.0
	BA	31	16.1	45.2	29.0	51.6
	ZJ	33	9.09	45.5	6.06	28.8
	CL	32	3.13	68.8	21.9	56.3
	LS	31	0.00	12.9	58.1	64.5
L1035S	FH	35	0.00	20.0	0.00	10.0
	PX	38	5.26	31.6	0.00	15.8
	BA	31	16.1	9.68	0.00	4.84
	ZJ	33	9.09	36.4	3.03	21.2
	CL	32	3.13	6.25	0.00	3.13
	LS	31	0.00	25.8	3.23	16.1
E1901D	FH	32	34.4	50.0	15.6	40.6
	PX	31	38.7	58.1	3.22	32.3
	BA	34	55.9	44.1	0.00	22.1
	ZJ	30	83.3	16.7	0.00	8.33
	CL	22	40.9	59.1	0.00	29.6
	LS	38	34.2	41.1	23.7	44.7

(“S” = the amino acid sequence of the sodium channel gene listed in GenBank (Accession No.: AB453977.1). “R” = mutant variants of “S”. “SS” = “S” homozygotes, “RS” = heterozygotes, and “RR” = “R” homozygotes).

^1^SS frequency = SS ÷ (SS + RS + RR) × 100%; ^2^RS frequency = RS ÷ (SS + RS + RR) × 100%.

^3^RR frequency = RR ÷ (SS + RS + RR) × 100%; ^4^R frequency = (RR + 1/2RS) ÷ (SS + RS + RR) × 100%.

### Analysis of genetic linkage between mutations

The results of HWE were showed in Tables 4. A significant (P > 0.05) heterozygote deficit was detected in only one (the LS) population, but there was evidence of heterozygote excess in some populations. The frequency of four mutations (R436K, M943V, I973T, and L1035F/S) significantly deviated from the HWE (P < 0.01) across all populations.

**Table 4:** **P-value of HWE test for heterozygote deficiency and excess confirmed at six loci in six** ***Cx. p. quinquefasciatus*** **populations**

Mutations	FH		PX		BA		ZJ		CL		LS		All populations
	Def.	Exc.	Def.	Exc.	Def.	Exc.	Def.	Exc.	Def.	Exc.	Def.	Exc.	P-value
V250M	0.6638	0.8045	0.8784	0.3797	0.5647	0.8103	1.0000	0.0729	1.0000	0.7097	0.0144	0.9992	0.3499
R436K	1.0000	0.0018	1.0000	0.0018	1.0000	0.0115	1.0000	0.0000	1.0000	0.0008	1.0000	0.0017	<0.01
M943V	1.0000	0.0060	1.0000	0.0218	1.0000	0.0047	1.0000	0.0611	1.0000	0.0020	1.0000	0.0001	<0.01
I973T	1.0000	0.0523	1.0000	0.0218	1.0000	0.0026	1.0000	0.0904	1.0000	0.0175	1.0000	0.0050	<0.01
L1035F/S	0.4900	0.5000	0.4186	0.5748	0.5859	0.4458	0.9620	0.0349	0.9990	0.0012	0.2784	0.7220	<0.01
E1901D	1.0000	0.0553	0.9902	0.0881	1.0000	0.3579	1.0000	0.8282	1.0000	0.2919	0.4961	0.7498	0.3615

The results of analysis of linkage disequilibrium between mutations are shown in Table 5. Significant linkage disequilibrium was found between M943V and I973T (P < 0.01). The L1035F/S mutation could also be linked with the M943V and I973T mutations because the *P-*values for these combinations are only slightly above 0.05. And sequencing data confirms the existence of linkage in some mosquitoes.

**Table 5:** **Chi-squared test results showed linkage between pairs of loci across six populations of** ***Cx. p. quinquefasciatus*** **(Fisher’s method)**

Locus pair	χ2	df	***P-*** value
V250M & R436K	0.0000	2	1.0000
V250M & M943V	10.307	12	0.5890
R436K & M943V	0.0000	2	1.0000
V250M & I973T	13.680	12	0.3216
R436K & I973T	0.0000	2	1.0000
M943V & I973T	Infinity	12	<0.01
V250M & L1035F/S	13.758	12	0.3164
R436K & L1035F/S	1.1216	2	0.5707
M943V & L1035F/S	20.486	12	0.0584
I973T & L1035F/S	20.864	12	0.0524
V250M & E1901D	12.551	12	0.4025
R436K & E1901D	0.0000	2	1.0000
M943V & E1901D	8.3050	12	0.7609
I973T & E1901D	6.0710	12	6.0710
L1035F/S & E1901D	16.813	12	0.1568

### Correlation between resistance and mutation frequencies

**Figure 4:**
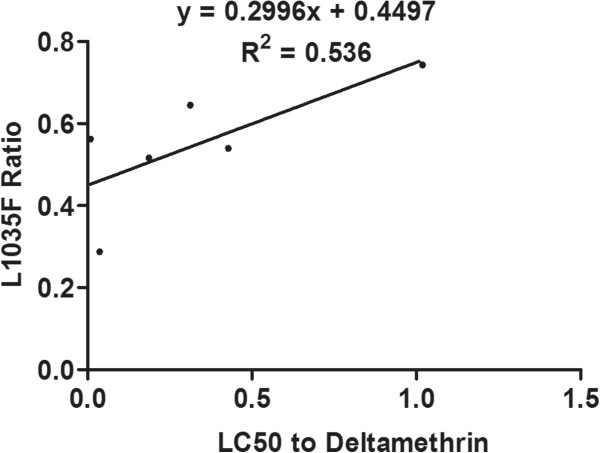
**Linear regression of the relationship between the frequency of the L1035F mutation and deltamethrin resistance.**

**Figure 5:**
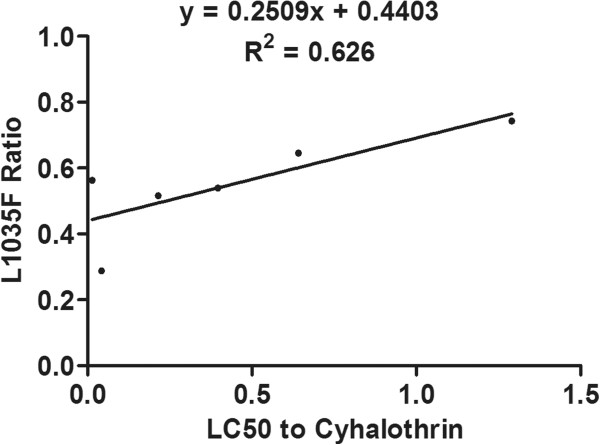
**Linear regression of the relationship between the frequency of the L1035F mutation and cyhalothrin resistance.**

**Figure 6:**
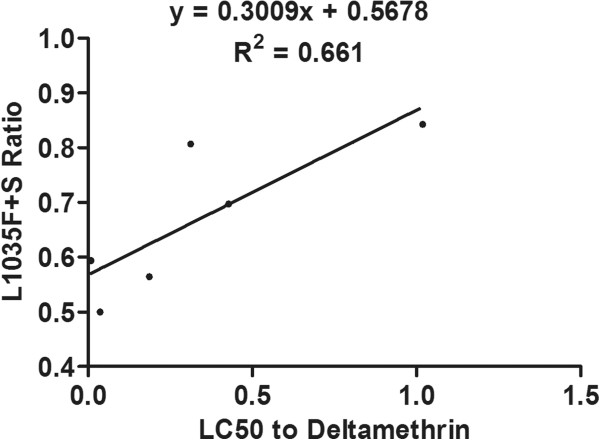
**Linear regression of the relationship between the frequency of the L1035 F + S mutation and deltamethrin resistance.**

**Figure 7:**
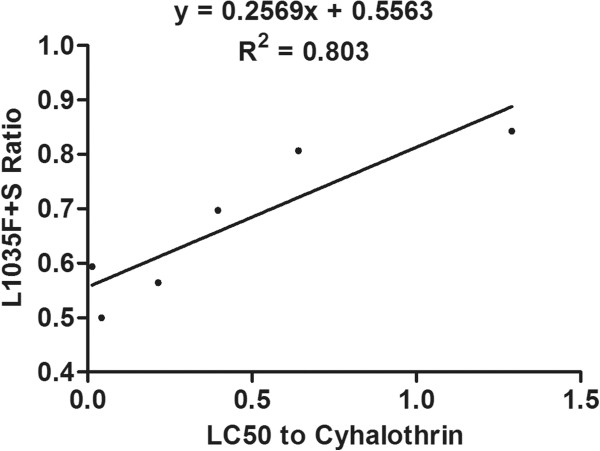
**Linear regression of the relationship between the frequency of the L1035 F + S mutation and cyhalothrin resistance.**

**Figure 8:**
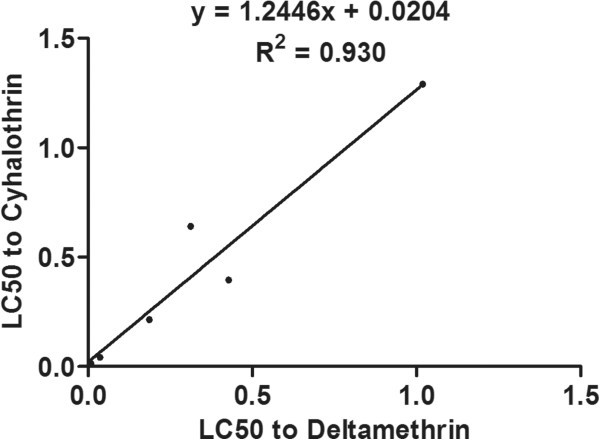
**Linear regression of the relationship between deltamethrin and cyhalothrin resistance (LC**_**50**_ **) in** ***Cx. p. quinquefasciatus*** **.**

The degree and significance of correlation between resistance to deltamethrin and cyhalothrin and the frequency of each mutation is shown in Table 6. The frequency of the L1035F mutation was significantly correlated with resistance to both deltamethrin (R^2^ = 0.536, P = 0.049) and cyhalothrin (R^2^ = 0.626, P = 0.030), and the combined frequency of the L1035F and L1035S mutations was significantly correlated with resistance to deltamethrin (R^2^ = 0.661, *P* = 0.025), and even more significantly correlated with resistance to cyhalothrin (R^2^ = 0.803, *P* = 0.008) (Figures 4, 5, 6 and 7). Frequencies of all other mutations were not correlated with either deltamethrin or cyhalothrin resistance. Significant cross-resistance between the two insecticides was also detected (R^2^ = 0.930, *P* < 0.01) (Figure 8).

**Table 6:** **Correlations calculated between frequencies of sodium channel gene mutations in** ***Cx. p. quinquefasciatus*** **and resistance to deltamethrin and cyhalothrin**

Mutations	Insecticide	R (95% CL)	R ^2^	***P-*** value (α = 0.05)
V250M	deltamethrin	0.054(-0.793,0.829)	0.003	0.460
	cyhalothrin	-0.046(-0.827,0.795)	0.002	0.465
M973V	deltamethrin	0.022(-0.804,0.819)	0.001	0.483
	cyhalothrin	0.235(-0.713,0.879)	0.055	0.327
I973T	deltamethrin	-0.240(-0.880,0.710)	0.058	0.323
	cyhalothrin	-0.158(-0.859,0.750)	0.025	0.383
L1035F	deltamethrin	0.732(-0.197,0.968)	0.536	0.049
	cyhalothrin	0.791(-0.057,0.976)	0.626	0.030
L1035S	deltamethrin	0.007( -0.809,0.814)	0.000	0.495
	cyhalothrin	0.040( -0.797,0.825)	0.002	0.470
L1035F + L1035S	deltamethrin	0.813( 0.005,0.979)	0.661	0.025
	cyhalothrin	0.896( 0.309,0.989)	0.803	0.008
E1901D	deltamethrin	0.609(-0.401,0.951)	0.371	0.100
	cyhalothrin	0.710(-0.240,0.965)	0.504	0.057

CL = confidence limits.

## Discussion

Insecticides have been used to control mosquitoes for more than half a century. Their indiscriminate use has, however, resulted in high levels of insecticide resistance in many mosquito species [8–10]. We tested the resistance of six Chinese *Cx. p. quinquefasciatus* populations to deltamethrin and cyhalothrin. Our results show that, compared to a susceptible laboratory strain, these six populations displayed a 9- to 1019-fold resistance to deltamethrin, and a 14- to 1291-fold resistance to cyhalothrin. The high resistance of the FH population may be related to its breeding habitat. This population frequently breeds in pools of water in peasant courtyards that are often polluted by insecticides.

The frequent use of insecticides has created an intense selection pressure for traits that confer resistance to them, such as changes in behavior, epidermal structure, metabolic enzymes and target-site mutations. Resistance may be conferred by the development of one, or more, of these traits. Osta et al. found that the dramatic reduction in the frequency of the G119S mutation in *Cx pipiens* mosquitoes was probably due to the increased use of pyrethroids over organosphosphate insecticides [35]. Therefore, alternating between different kinds of insecticide is one way of reducing the development of resistance to any one type.

We identified seven point mutations at six loci and eleven alternative splices in the sodium channel gene of Chinese *Cx. p. quinquefasciatus*. The Hardy–Weinberg equilibrium (HWE) describes the theoretical frequency of two alleles at the same locus, in the absence of mutation and selection, in an indefinitely large population with discrete generations after one generation of random mating [36]. The results of HWE tests suggested that mutations at four of these six loci significantly deviated from the HWE (P < 0.05). Among these, the R436K mutation occurred at a frequency of nearly 50% in all six populations and there was an excess of heterozygotes with this mutation (P < 0.05). How this mutation developed and its role, if any, in pesticide resistance requires further investigation.

Mutations at three other loci (M943, I973 and L1035) also deviated from the HWE, and some populations had an excess of heterozygotes with these mutations. L1014 (L1035 according to our sequencing) is a classical mutation associated with the use of pyrethroid insecticides. One reason why the previous three mutations deviate from the HWE may be because a long period of selection has led to them becoming fixed. Mutations at two other loci (V250M and E1901D) did not deviate from the HWE.

We found evidence of significant linkage between the M943V and I973T mutations (P < 0.01) and *P-*values for a Chi-squared test of association between L1014F/S and M943V and I973T were also only slightly above 0.05. Our sequencing data support linkage between these mutations in some mosquitoes but this requires further confirmation.

The V250M mutation is the first to be discovered in the *Cx. p. quinquefasciatus* sodium channel gene’s IS4 domain, in which mutations rarely occur. The frequency of this mutation was not correlated with resistance to either deltamethrin or cyhalothrin so further research is required to determine its function, if any, in pesticide resistance.

Our results provide the first confirmation of the R436K mutation, located between the first and second homology domains of the sodium channel gene near the IS6 domain, in *Cx. p. quinquefasciatus*. The E434K mutation was first identified near the IS6 domain of the sodium channel gene in the German cockroach. This mutation is associated with two other mutations, C764R and L993F (L1014F), that are closely linked to resistance to the pyrethroid insecticide cypermethrin [37]. We did not find any correlation between the frequency of this mutation and pyrethroid resistance. However, almost all individuals with this mutation were heterozygotes and its frequency was almost 50% across all populations. The reasons for its prevalence require further investigation.

The M943V and I973T mutations are located at the junctions between IIS4 and IIS5, and between IIS5 and IIS6, in the sodium channel gene and were significantly linked (P < 0.01). The M943V mutation is only three amino acids from the M918T site (the amino acid number in the housefly sodium channel gene, GenBank Accession No.: X96668). The M918T mutation is usually associated with the L1014F mutation in which case it confers “super-kdr” resistance. However, some studies have reported super-kdr resistance in the absence of the L1014F mutation. For example, Benjamin et al. found that the M918T mutation greatly increased the resistance of *Tetranychus evansi* to pyrethroid insecticides in the absence of the L1014F mutation [38].

The I874M mutation (corresponding to the M918 position in the housefly) in mammals can result in a 100-fold desensitization of the sodium channel [39]. Another study identified a methionine to valine replacement at this position in the *Bemisia tabaci* para-sodium channel gene that was unrelated to resistance [40]. We also detected a methionine to valine replacement at the fourth amino acid after the M918 position in all six *Cx. p. quinquefasciatus* populations that was not correlated with deltamethrin or cyhalothrin resistance. How this mutation arose, its connection with the M918 point mutation, linkage with the I973T mutation and role, if any, in resistance, requires further investigation.

L1035 is the site of the classic knockdown resistance mutation L1014 (amino acid number in the housefly sodium channel gene, GenBank Accession No.: X96668). It is located in the IIS6 domain of sodium channel, and has been relatively well researched. Subsequent studies have identified mutations associated with knockdown resistance in a variety of insects, for example, *Aphis gossypii*[15], the German cockroach [16], *Myzus persicae*[17]*Triatoma infestans*[21], *Anopheles gambiae*, *Anopheles stephensi* and *Anopheles arabiensis*[22]. In addition, recent studies have that the L1014S mutation is associated with permethrin, cypermethrin, cyhalothrin and DDT resistance in *Anopheles sinensis*, *An. vagus* and *An. peditaeniatus*, [41]; with deltamethrin resistance in *Cx p. pallens*[42], and with permethrin and DDT resistance in *Anopheles gambiae*[13, 43].

Another study found a new mutation, V1010L, that is closely linked to the L1014S mutation in *Anopheles culicifacies* but did not mention if it was associated with resistance [25]. As previously mentioned, the co-occurrence of the L1014F and M918T mutations can lead to super-kdr resistance in many insects. For example, these two mutations are closely related to cyhalothrin and permethrin resistance in *Haematobia irritans*[29]; and resistance to a suite (permethrin, bifenthrin, tefluthrin, deltamethrin, cypermethrin, cyhalothrin and fluvalinate) of pyrethoid insecticides in *Myzus persicae*[30]. No one has so far, however, detected super-kdr resistance in a mosquito species.

In *Drosophila melanogaster*, the L1014F, M918T and T929I mutations affect the sodium channel currents in the presence of deltamethrin and permethrin, suggesting that these mutations confer resistance to those pesticides [44]. Other authors have found the above three mutations in the sodium channel gene of pyrethroid-resistant *Thrips tabaci*[45] and identified the L1014H, L1014C and L1014W mutations [26–28, 46, 47].

Studies have showed mutations in L1014 could lead to a reduction in susceptibility to a variety of pyrethroids and a decay of tail current. And then lead to develop resistance [48, 49]. We found that the L1035F and L1035S mutations were present in all six *Cx. p. quinquefasciatus* populations we sampled and that these mutations were significantly correlated with deltamethrin and cyhalothrin resistance. This may be related to the frequent use of these pesticides at our sampling sites. In addition, we found that the frequency of the leucine to serine replacement in all six populations was lower than that of the L1035F mutation. The combined frequency of the L1035F and S mutations was highly correlated with resistance, especially cyhalothrin resistance (R^2^ = 0.803, P < 0.01), which suggests that mosquitoes in the sampled populations have developed additional mutations except the L1035F mutation in response to strong selection pressure.

This study was the first to detect the E1901D mutation, located on the sodium channel C-terminal tail, in *Cx. p. quinquefasciatus*. There has, so far, been relatively little research on this fragment but it appears to be unrelated to deltamethrin and cyhalothrin resistance. Determining the function of such mutations will require further study.

We confirmed eleven alternative splices in the sodium channel gene of *Cx. p. quinquefasciatus*. These reflect dynamic change and higher level reassembly of genetic information that can greatly increase the richness of the coding sequence without changing genomic DNA [50]. Liu et al. (2012) found thirteen different sodium channel variants in *Cx. p. quinquefasciatus* and demonstrated that this alternative splicing was related to pyrethroid resistance [51]. The eleven alternative splices we found differed from those found by Liu et al. They may have some significance in the rich diversity of the sodium channel protein, but further research is required to determine their role, if any, in pyrethroid resistance.

## Conclusion

In the study, seven amino acid alterations were found (V250M, R436K, M943V, I973T, L1035F, L1035S and E1901D) in the sodium channel gene of *Cx. p. quinquefasciatus* from six field populations in China. Among them, the L1035F and L1035S mutations’ frequencies were associated with resistance to deltamethrin and cyhalothrin. And the M943V and I973T mutations were significant linkage with each other in the sodium channel gene. Identifying these mutations may be of practical benefit to the development of pesticide resistance management programs.
